# The influence of the host microbiome on 3,4-methylenedioxymethamphetamine (MDMA)-induced hyperthermia and vice versa

**DOI:** 10.1038/s41598-019-40803-3

**Published:** 2019-03-13

**Authors:** Emily A. Ridge, Sudhan Pachhain, Sayantan Roy Choudhury, Sara R. Bodnar, Ray A. Larsen, Vipaporn Phuntumart, Jon E. Sprague

**Affiliations:** 1 The Ohio Attorney General’s Center for the Future of Forensic Science, Bowling Green State University, Bowling Green, OH 43403 USA; 2 The Department of Biological Sciences, Bowling Green State University, Bowling Green, OH 43403 USA

## Abstract

Hyperthermia induced by 3,4-methylenedioxymethamphetamine (MDMA) can be life-threatening. Here, we investigate the role of the gut microbiome and TGR5 bile acid receptors in MDMA-mediated hyperthermia. Fourteen days prior to treatment with MDMA, male Sprague-Dawley rats were provided water or water treated with antibiotics. Animals that had received antibiotics displayed a reduction in gut bacteria and an attenuated hyperthermic response to MDMA. MDMA treated animals showed increased uncoupling protein 1 (*UCP1)* and *TGR5* expression levels in brown adipose tissue and skeletal muscle while increased expression of UCP3 was observed only in skeletal muscle. Antibiotics prior to MDMA administration significantly blunted these increases in gene expression. Furthermore, inhibition of the TGR5 receptor with triamterene or of deiodinase II downstream of the TGR5 receptor with iopanoic acid also resulted in the attenuation of MDMA-induced hyperthermia. MDMA-treatment enriched the relative proportion of a *Proteus mirabilis* strain in the ceca of animals not pre-treated with antibiotics. These findings suggest a contributing role for the gut microbiota in MDMA-mediated hyperthermia and that MDMA treatment can trigger a rapid remodeling of the composition of the gut microbiome.

## Introduction

3,4-Methylenedioxymethamphetamine (MDMA) is a synthetic sympathomimetic agent more commonly known as “Ecstasy” or “Molly.” MDMA induction of hyperthermia involves both central and peripheral triggers. Centrally, MDMA results in the activation of dopaminergic^1^ and serotonergic^2^ receptors in thermoregulatory circuits in the hypothalamus^3–5^; ultimately activating peripheral mediators of heat generation. In the periphery, MDMA-mediated increases in norepinephrine binding to the *α*_1_-adrenergeric receptor in vascular smooth muscle, results in vasoconstriction and attenuated heat dissipation^6,7^. MDMA-induced norepinephrine release also triggers thermogenesis^8^ by two routes; each mediated by signaling through β-adrenergic receptors in brown and white adipose tissue. Norepinephrine mediated activation of β-adrenergic receptor increases the expression of the *UCP1* gene, leading to the production of the uncoupling protein UCP1^9^. β-adrenergic receptor activation further induces lipolysis, with the resultant release of free fatty acids (FFA) from brown adipose tissue (BAT) and white adipose tissue (WAT) with subsequent transport of FFA into skeletal muscle mitochondria to serve as ligand activators for UCP-facilitated proton leak^10,11^. When activated, UCPs dissipate the proton gradient across the inner mitochondrial membrane, resulting in increased proton conductance and the release of energy as heat^12^.

BAT-mediated thermogenesis is an important component in mammalian thermal homeostasis. Dependent upon stored metabolic fuels, BAT-mediated thermogenesis is modulated by a variety of signals reflecting the metabolic and stored fuel status of the organism^13^. Bile acids provide one such signal, increasing energy expenditure in a UCP-dependent fashion in BAT and skeletal muscle^14^. Using the G-protein coupled receptor TGR5, bile acids stimulate the production of cyclic AMP, inducing 2-iodothyronine deiodinase (D2) to convert local thyroxine (T_4_) into 3,5,3-tri-iodothyronine (T_3_). T_3_ in turn stimulates glucose metabolism and lipolysis, fueling thermogenesis^15^. Binding of bile acids to TGR5 in intestinal cells stimulates the production of glucagon-like peptide 1 (GLP1)^16^, an insulinotropic hormone that stimulates BAT thermogenesis^17^.

Bile acids are produced by hepatocytes and secreted into the duodenum where they function in the absorption of lipids and lipid soluble molecules. The intestinal microbiome actively modulates the size and composition of the bile acid pool^18^. The farnesoid X receptor (FXR) provides for negative feedback regulation of bile acid synthesis^19^. Tauro-conjugated muricholic acids act as FXR antagonists, limiting hepatic bile acid synthesis under normal conditions; however, in germ-free^20^ or antibiotic treated mice^21^, tauro-conjugates are not modified by microbial activity, resulting in a much larger bile acid pool, indicating that alterations in gut microbiome can alter bile acid composition^22^. Interestingly, mice undergo dramatic remodeling of their gut microbiota when adapting to cold temperatures with accompanying changes in BAT tissue and browning of white adipose tissue. These tissue changes were transferable with microbiota transplantation into germ-free mice^23^. Based on these studies and previous knowledge of UCP regulation of MDMA-induced hyperthermia, we hypothesized that the actions of some members of the intestinal microbiota might influence the sympathomimetic-induced thermogenic response to MDMA. Because of the role of bile acids in UCP regulation and role of the intestinal microbiome in regulating the size and composition of the bile acid pool^18^, we further tested the role of the TGR5 receptor^24^ and D2^25^ in MDMA-mediated hyperthermia through their inhibition with triamterene and iopanoic acid respectively. The results of this study support this hypothesis and further suggests that MDMA can in turn trigger a rapid remodeling of the microbiota composition in at least some intestinal compartments.

## Results

### Quantification of Intestinal Bacteria by qPCR

**Figure 1:**
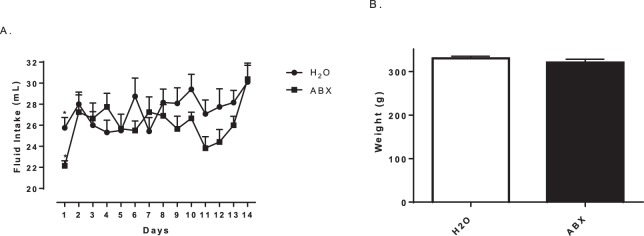
Daily fluid intake for both antibiotic-treated (ABX) and control (H_2_O) groups (**A**). Mean body weight for both antibiotic-treated and control groups (**B**). * indicates significantly different from all other treatment groups (*p* = 0.0019). Each value is the mean ± SEM (n = 12).

**Figure 2:**
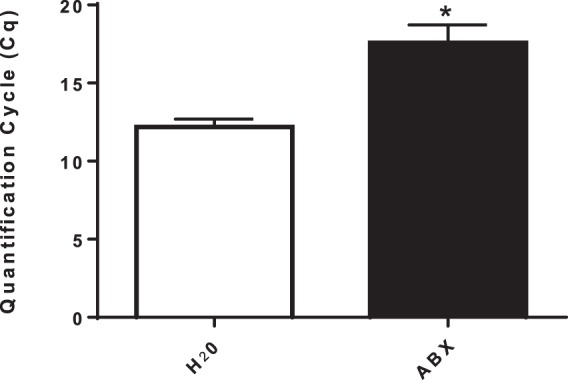
Quantification of cecal bacteria by qPCR. Quantification cycles (Cq) values of the 16S rRNA gene using 200 ng of a pool of DNA extracted from twelve animals per treatment. All qPCR assays were run in triplicate including no template negative controls. * indicates significant difference between treatment groups (*p* = 0.0016). Each value is the mean ± SEM (n = 12).

To determine if changes in intestinal microbiota influence the thermogenic response to MDMA, animals were provided a cocktail of antibiotics (ABX): bacitracin, neomycin, and vancomycin, via their drinking water, for 14 days before MDMA treatment. With the exception of the first day of exposure to the antibiotics (Fig. 1A**-** two-tailed t-test: *p* = 0.0019; t = 3.535), daily fluid intake and body weight (Fig. 1B**-** two-tailed t-test: *p* = 0.2144; t = 1.278) did not differ between groups over the course of the experiment. To ensure the ability of mixed ABX in reducing gut bacteria, total DNA was purified from 200 mg of the cecum of each animal and they were pooled together, followed by qPCR analysis using 200 ng of pooled DNA and universal primers specific for the bacterial 16S rRNA gene. Based on the quantification cycles (Cq) values, a significant reduction in apparent bacterial number was evident in the ABX groups compared to the H_2_O control groups (Fig. 2**-** two-tailed t-test: *p* = 0.0016; t = 4.296). Prolonged treatment with antibiotics resulted in markedly enlarged ceca (data not shown), similar to the previous observations of others^26^.

### Analysis of Cultivatable Cecal Bacteria

**Figure 3:**
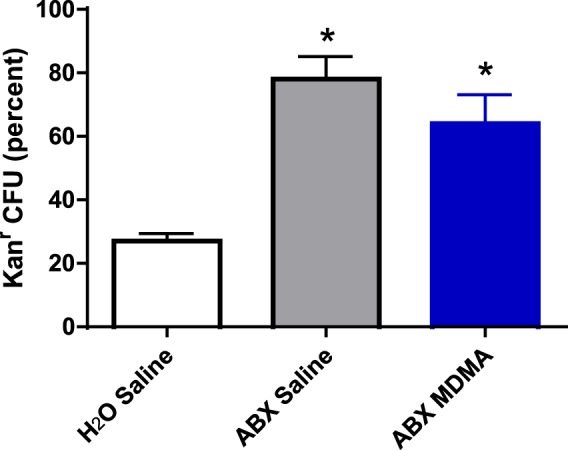
Relative proportions of kanamycin-tolerant culturable bacteria recovered in cecal contents. * indicates significantly different from H_2_O control group (p = 0.006) Each value is the mean ± SEM (n = 6).

qPCR analysis (Fig. 2) established that ABX treatment reduced bacterial numbers in the cecum relative to untreated controls, evident by the increased Cq value for the ABX-treated animals (Fig. 2). Our working assumption was that ABX treatment would alter the composition of the cecal microbiota by differentially selecting against antibiotic sensitive bacterial strains. To determine if this occurred, we evaluated kanamycin resistance in cecal samples harvested at necropsy from each of the four groups. Resistance to kanamycin would be indicative of the selection of strains capable of producing aminoglycoside modifying enzymes by the ABX regimen. Comparison of the relative proportions of kanamycin resistant culturable bacterial verified that such alterations did occur. For the six saline-challenged animals that received no antibiotics (H_2_O control), only 15–48% (mean = 27%) of the culturable cecal bacteria grew in the presence of kanamycin, whereas for the six saline-challenged ABX treated animals (ABX control) 53–100% (mean = 78%) of the culturable cecal bacteria grew in the presence of kanamycin. A similar proportion of culturable cecal bacteria (46–83%; mean = 64%) able to grow in the presence of kanamycin were found in the six MDMA-challenged ABX treated animals (Fig. 3-One way ANOVA with Student-Newman-Keuls post hoc test: F_(2,15)_ = 12.823, p = 0.006).

**Figure 4:**
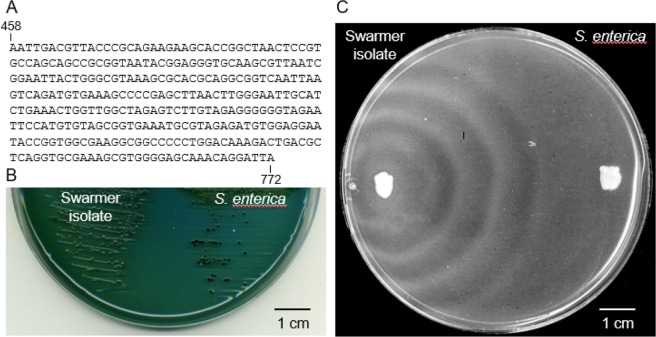
Identification of swarming bacteria. (**A**) Consensus sequence representing eight swarmer isolates, corresponding and identical to bases 458–772 of rRNA gene of *P*. *mirabilis* AR_0029 (Genebank CPO29725) and numerous other isolates and *S*. *enterica* serovar Typhimurium (Genebank MH356711). (**B**) Comparison of swarmer isolate and *S*. *enterica* serovar Typhimurium grown on Hektoen enteric agar for 18 hrs at 37 °C. Scale is indicated by the 1 cm bar at the lower right of the figure. (**C**) Comparison of swarmer isolate and *S*. *enterica* serovar Typhimurium grown on LB agar for 18 hrs at 37 °C. Contrast in this image was increased to enhance visualization of the swarming pattern. Scale is indicated by the 1 cm bar at the lower right of the figure.

For the six non-ABX-treated MDMA-challenged animals (H_2_O MDMA), the proportion of kanamycin-resistant culturable bacteria could not be determined, as the colonies were obscured by the presence of swarming bacteria on plates lacking kanamycin. Bacterial swarming on a nutrient-rich medium is a phenotype associated with the genus *Proteus*, a common inhabitant of mammalian digestive systems^27^. The swarmers were subsequently isolated as single colonies on MacConkey agar, which contains bile salts that inhibit swarming behavior. Colony PCR of eight such isolates using primers specific for 16S rRNA produced amplimers corresponding with a sequence to a 314-base pair region of the 16S rRNA genes (GeneBank accession number MK033607) that is shared by both *Proteus mirabilis* and certain isolates of *Salmonella enterica*, including a serovar Typhimurium strain, all with 100% sequence identity and minimum E-values of 2.30 E^−158^ (Fig. 4A). Isolates were compared with a well-documented strain of *S*. *enterica* serovar Typhimurium (ATCC 19585) by plating on another bile salt-containing medium, Hektoen enteric (HE) agar, that distinguishes salmonella from other enteric bacteria based on the production of hydrogen sulfide, as visualized by the formation of black ferric sulfate precipitates in the inner region of the colony. Such precipitates were not evident in swarmer isolates grown on HE agar, but were apparent for the *S*. *enterica* strain (Fig. 4B). Swarmer isolates were re-plated onto a nutrient rich medium (LB) where swarmer behavior was again evident, in contrast to *S*. *enterica*, which did not swarm (Fig. 4C). Collectively, these data suggest that the swarming bacteria are *Proteus mirabilis*.

### Effects of Gut Microbiota on MDMA-Induced Hyperthermia

**Figure 5:**
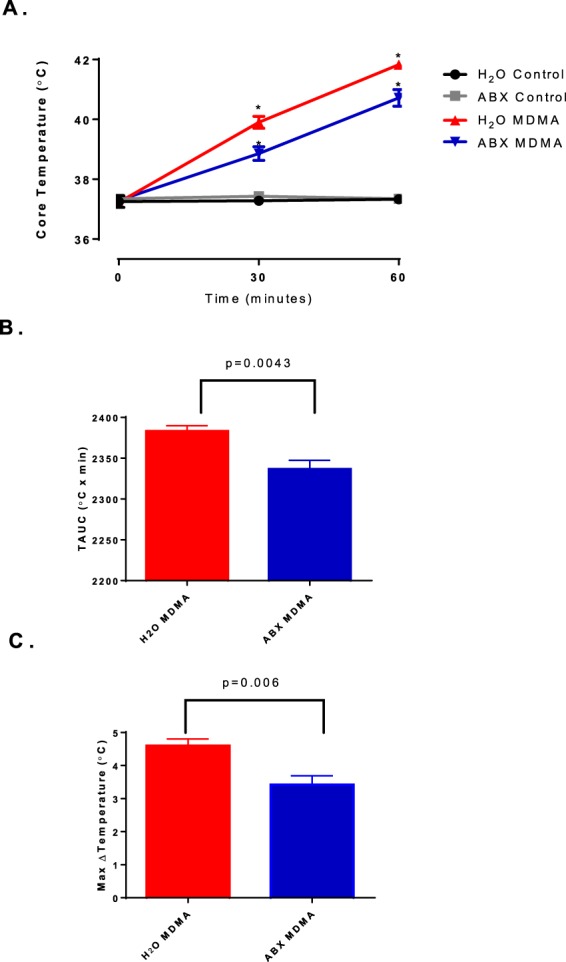
Changes in core temperature following the administration of MDMA (**A**). Temperature-area under the curve (TAUC) following the administration of MDMA (**B**). Maximum change in temperature following the administration of MDMA (**C**). * indicates significant difference from all other treatment groups (p < 0.05).

MDMA significantly increased core body temperature in both the antibiotic (ABX) and water (H_2_O) experimental groups at the 30- and 60-minute time points as compared to control groups (Fig. 5A-One way ANOVA with Dunnett’s post hoc: F_(2,15)_ = 65.14, p < 0.0001). The ABX MDMA group showed an attenuated thermogenic response compared to that of the H_2_O MDMA group (Fig. 5A-One way ANOVA with Student-Newman-Keuls post hoc between time points: F_(3,20)_ = 177.35, p < 0.0001). Calculation of the temperature area under the curve (TAUC) provided additional evidence for the diminished thermogenic responses of the ABX MDMA group relative to that of the H_2_O MDMA group (Fig. 5B**-** two-tailed t-test: *p* = 0.0043; t = 3.67). ABX pretreatment before MDMA significantly attenuated the maximal change in core temperature (Fig. 5C**-** two-tailed t-test: *p* = 0.006; t = 3.47). To exclude the possibility that the effects of the oral antibiotic treatment on MDMA-induced hyperthermia were due to pharmacodynamic interactions between MDMA and the antibiotics, a second experiment was performed, with animals injected with a single intraperitoneal dose of antibiotics (1.67 mg/kg vancomycin, 20 mg/kg neomycin, and 293 U/kg bacitracin in PBS) thirty minutes prior to treatment with MDMA. These animals did not display an attenuated thermogenic response to MDMA. Instead, these animals displayed a similar maximum change in temperature as the control group (data not shown**-** two-tailed t-test: *p* = 0.4525; t = 0.781), supporting the interpretation that components of the microbiota, not the antibiotics, were responsible for the attenuated thermogenic response to MDMA in the ABX MDMA group.

### MDMA Treatment Results in Alteration of the Expression of Genes Associated with Hyperthermia

**Figure 6:**
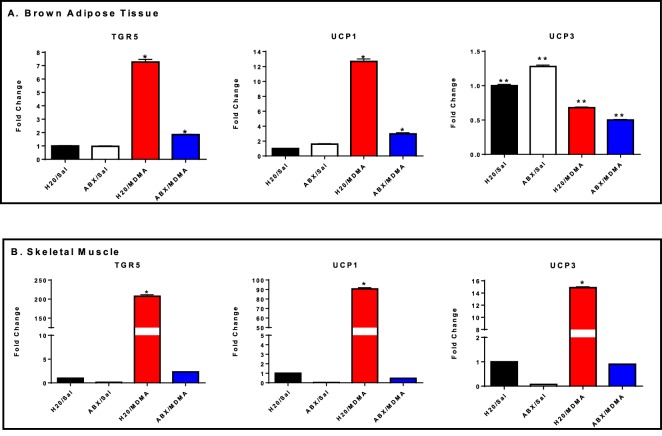
qPCR gene expression analysis of TGR5, UCP1 and UCP3 in (**A**) BAT and (**B**) skeletal muscle. * significantly different from all other treatment groups (p < 0.01). ** significantly different from all other treatment groups (p < 0.001). Each value is the mean ± SEM (n = 6).

To assess the molecular mechanisms underpinning MDMA-mediated hyperthermia, expression of *UCP1*, *UCP3* and *TGR5* was examined in BAT and skeletal muscle by qRT-PCR. Results were normalized to β-actin. In BAT, MDMA treatment showed elevated expression of *UCP1* (Fig. 6A-One way ANOVA with Student-Newman-Keuls post hoc test: F_(3,8)_ = 849.93, p = 0.0001) and *TGR5* (Fig. 6A-One way ANOVA with Student-Newman-Keuls post hoc test: F_(3,8)_ = 2373, p = 0.0001) while the expression of *UCP3* was reduced compared to controls (Fig. 6A-One way ANOVA with Student-Newman-Keuls post hoc test: F_(3,8)_ = 339.12, p = 0.0001). In skeletal muscle, MDMA increased the expression of *UCP1* (Fig. 6B-One way ANOVA with Student-Newman-Keuls post hoc test: F_(2,5)_ = 3857, p = 0.0001), *UCP3* (Fig. 6B-One way ANOVA with Student-Newman-Keuls post hoc test: F_(3,8)_ = 10872, p = 0.0001) and *TGR5* (Fig. 6B-One way ANOVA with Student-Newman-Keuls post hoc test: F_(3,8)_ = 2696, p = 0.0001). Treatment with ABX for 14 days prior to MDMA challenge down-regulated the expression of these genes in both BAT and skeletal muscle compared to H_2_O MDMA group.

### Effect of triamterene (TM) on MDMA-induced hyperthermia

**Figure 7:**
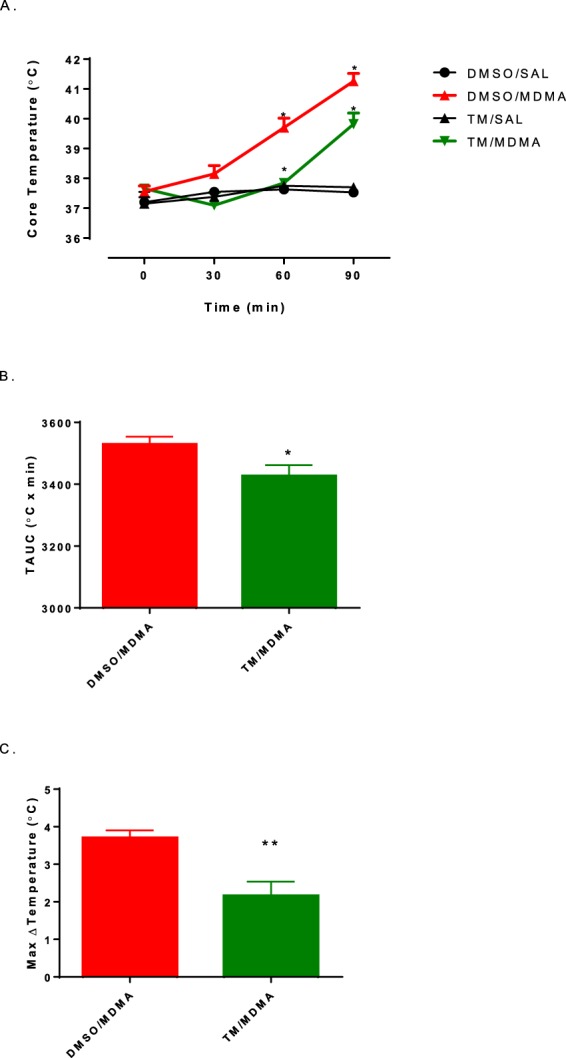
Effect of triamterene pretreatment on MDMA-induced hyperthermia. (**A**) The effects of triamterene pretreatment 30 minutes prior to MDMA on core body temperature over a 90-minute time interval. [Each value is the mean ± SEM (n = 6). * indicates significantly different between treatment groups (p < 0.05)]. (**B**) Temperature Area Under the Curve (TAUC) following MDMA treatment 30 minutes after triamterene pretreatment. Each value is the mean ± SEM (n = 6). * indicates significantly different between treatment groups (p < 0.05). (**C**) Max Change in Temperature (ΔTmax) following treatment of MDMA 30 minutes prior to triamterene pretreatment. Each value is the mean ± SEM (n = 6). ** indicates significantly different between treatment groups (p < 0.01).

In both the vehicle (VEH)/MDMA and TM/MDMA groups, MDMA administration resulted in a significantly higher core body temperature than the other treatment groups at the 90-minute time point (Fig. 7A-One way ANOVA with Student-Newman-Keuls post hoc between time points: F_(3,19)_ = 64.05, p = 0.0001). Animals treated with TM 30 minutes prior to MDMA demonstrated an attenuated thermogenic response when compared to the VEH/MDMA group (Fig. 7A-One way ANOVA with Student-Newman-Keuls post hoc between time points: F_(3,18)_ = 23.34, p = 0.0001). TAUC analysis for the VEH/MDMA group was significantly greater compared to the TM/MDMA group (Fig. 7B**-** two-tailed t-test: *p* = 0.04; t = 2.43). MDMA induced a hyperthermic response that resulted in a maximal temperature change (ΔTmax) of 3.7 ± 0.2 °C, while TM treatment prior to MDMA attenuated the hyperthermic response with a ΔTmax of 2.2 ± 0.4 °C (Fig. 7C**-** two-tailed t-test: *p* = 0.0045; t = 3.763).

### Effect of iopanoic acid (IOP) on MDMA-induced hyperthermia

**Figure 8:**
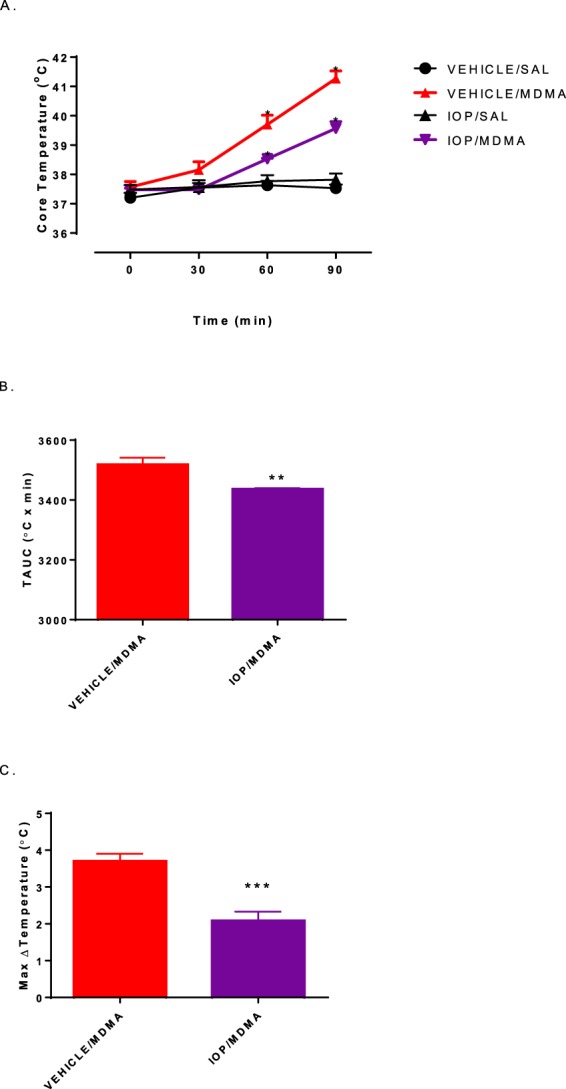
Effects of IOP pretreatment on MDMA-induced hyperthermia. (**A**) The effects of IOP pretreatment each day for 7 days and 30 minutes prior to MDMA on core body temperature over a 90-minute time interval. Each value is the mean ± SEM (n = 6). * indicates significantly different between treatment groups (p < 0.05). (**B**) Temperature Area Under the Curve (TAUC) following MDMA treatment 30 minutes after iopanoic acid pretreatment. Each value is the mean ± SEM (n = 6). ** indicates significantly different between treatment groups (p < 0.01). (**C**) Max Change in Temperature (ΔTmax) following treatment of MDMA 30 minutes after iopanoic acid pretreatment. Each value is the mean ± SEM (n = 6). *** indicates significantly different between treatment groups (p < 0.001).

In both the VEH/MDMA and IOP/MDMA groups, MDMA administration resulted in a significantly higher core body temperature than the other treatment groups at both the 60- and 90-minute time points. Additionally, pretreatment with IOP significantly attenuated MDMA-induced hyperthermia at the 60- and 90-minute time points when compared to the VEH/MDMA group (Fig. 8A-One way ANOVA with Student-Newman-Keuls post hoc between time points: F_(3,20)_ = 64.67, p = 0.0001). Furthermore, the TAUC for VEH/MDMA was significantly greater than IOP/MDMA (Fig. 8B**-** two-tailed t-test: *p* = 0.005; t = 3.562). Finally, the IOP/MDMA treatment resulted in a ΔTmax of 2.1 ± 0.2 °C, which was significantly less than that of VEH/MDMA ΔTmax of 3.7 ± 0.5 (Fig. 8C**-** two-tailed t-test: *p* = 0.0005; t = 5.072).

## Discussion

Several studies have found that microbiome-generated heat contributes to the maintenance of body temperature in animals (for a review see^28^). Oral treatment of rabbits^29^ and rodents^30,31^ with non-absorbable antibiotics lowers core body temperature. Recent findings using Brandt’s voles suggest that changes in core body temperature changes the host gut microbial community, which in turn regulates the host’s thermal homeostasis^32^. The present findings suggest that the microbiome-gut-brain axis may play a contributory role in the hyperthermia mediated by MDMA. ABX treatment for 14 days prior to MDMA treatment not only reduced the number and altered the composition of the cecal bacterial population, but also attenuated MDMA-induced hyperthermia. Recently, Althobailti *et al*.^33^, demonstrated that the β-lactam antibiotic, ceftriaxone, administered for three days following methamphetamine treatment blocked the methamphetamine-induced hyperthermia. Those authors did not examine the effects of ceftriaxone treatment on the gut microbiome, but linked the effects to ceftriaxone regulation of the central expression of the glutamate transporter. Intraperitoneal injection of minocycline for three days has been shown to attenuate the hyperthermic effects of MDMA^34^, but had no effect on body temperature when given for two days^35^. Previous studies by Rawls *et al*.^36^ found that ceftriaxone given for seven days prior to hyperthermic doses of morphine also blocked hyperthermia. They suggested a potential role for the glutamate transporter, but did not examine the effects of ceftriaxone on the gut microbiome. This present study is the first to examine the potential role of the bacterial component of the gut microbiome in the hyperthermia associated with drugs of abuse. Although the results of the present study demonstrate a potential relationship between MDMA-induced hyperthermia and the gut microbiome, they do not suggest that the microbiome is a major contributor to this hyperthermia in that the effects of the ABX treatment were slight yet significant.

We have recently ascertained that UCP1 and UCP3 play complementary roles in the onset (UCP1) and maintenance (UCP3) of sympathomimetic-induced hyperthermia^37^. In the present study, we observed an increase in *UCP1* expression in both BAT and skeletal muscle following MDMA challenge while *UCP3* expression increased only in skeletal muscle. ABX treatment blocked these changes in *UCP* expression levels, consistent with our previous studies regarding the role of UCP in MDMA-mediated hyperthermia^37^. The TGR5 receptor has been suggested to play a role in cold-induced thermogenesis through its regulation of the thyroid-catalyst protein deiodinase II (D2) and the ultimate regulation of *UCP* expression^14,38^. Similarly, in both BAT and skeletal muscle of our rat models, MDMA increased *TGR5* expression levels. Conversely, prior ABX treatment rendered *TGR5* gene expression refractory to this MDMA-induced change.

Recent studies have suggested that TM inhibits the TGR5 receptor^24^. Those authors reported that TM was able to dose-dependently inhibit the increase in glucose uptake mediated by TGR5 agonists in Chinese hamster ovary cells (CHO-K1 cells). Moreover, glucagon-like peptide-1 secretion and increased cAMP levels, which are induced by TGR5 activation, were both shown to be dose dependently reduced by TM^24^. Additional animal studies by the same researchers using streptozotocin-induced diabetic rats supported these findings, indicating that TM is an effective TGR5 antagonist^24^. In the present study, we demonstrate and confirm that TM functions as an antagonist of the TGR5 receptor to attenuate the hyperthermia mediated by MDMA. Additionally, the D2-inhibitor IOP significantly attenuated MDMA-mediated hyperthermic response. IOP was first shown to inhibit D2 activity and the enzymatic conversion of T4 to T3 by Larsen *et al*.^25^. T3 has been demonstrated to increase *UCP*1 and *UCP3* mRNA expression in BAT and skeletal muscle, respectively^39,40^. TGR5 receptor activation facilitates cAMP production that ultimately leads to an increase of D2 production^14^. The attenuated MDMA-mediated hyperthermic response seen after D2 inhibition is consistent with previous studies from our laboratory, demonstrating a role for thyroid hormone in the hyperthermia mediated by MDMA^6^.

This study suggests that the resident gut flora influences the physiological response of their host to the challenge of MDMA. The nature of this influence, and the specific members of the resident microbial community responsible, remains to be discerned, but clearly merits further consideration. Identifying mechanisms by which the microbiota modulate hyperthermic responses provides alternative strategies to dissect this and other complex regulatory pathologic processes. The fact that MDMA changed the composition of the gut microbiota, coupled with the observation that altering the gut microbiota with antibiotics modified the thermogenic response to MDMA in the rodent model, suggests a potential relationship between human gut microbiota and body temperature regulation of relevance to our understanding of MDMA-induced pathological thermogenesis in humans.

The hyperthermia mediated by sympathomimetic agents such as MDMA involves both peripheral and central mechanisms. The communication between the gut-brain-axis is complex and involves many of the same neurotransmitters that are targets for the pharmacological and toxicological actions of MDMA. For example, serotonin and norepinephrine play key roles in regulating the communication between gut-brain-axis. Subsequently, an association has been made between intestinal bacteria and the pathophysiology of CNS disorders such as autism, depression and substance-use disorders (for a review, see^41^). In the present study, we did not examine the potential link between the gut-brain-axis and studies in this area are therefore warranted.

This present study was optimized to provide for a consistent pathological response to afford greater precision to evaluate the potential of individual components (such as the composition of the gut microbiota) to enhance MDMA-induced thermogenesis. To this end, we used a 20 mg/kg dose of MDMA. This is the standard dose that our lab has used to induce MDA induced hyperthermia^6^. Additionally, toxicity studies with MDMA typically use a dosage range of 10 to 80 mg/kg (for a review, see^42^). Ambient temperature has been shown to influence the thermogenic response to amphetamines^4,43,44^. O’Shea *et al*.^45^ demonstrated that rats treated with MDMA in environmental temperatures of 30 °C or higher became hyperthermic. However, if the ambient temperature is reduced to 20–22 °C, MDMA mediates a hypothermic response^46^. In present study, we maintained an ambient temperature 24–26 °C. Finally, we had previously demonstrated that the amount of fat in the diet further enhanced the thermogenic response to MDMA^47^. We therefore fed the animals a 10% fat diet.

Intriguingly, this study found the presence of a *Proteus* population of sufficient relative number to obscure colony counting, but only in non-ABX animals treated with MDMA. This indicated that MDMA itself either directly or indirectly stimulates rapid alterations in the composition of the resident bacterial population of the cecum. This suggests the possibility that the repeated use of MDMA and similar compounds may result in long-term alterations in the composition of resident intestinal bacterial populations. This possibility and its potential consequences also merit further consideration.

## Methods

### Animals

Male Sprague-Dawley rats (284.6 ± 2.4 g, Envigo, Indianapolis, IN) were used. Animals were housed one per cage (21.0 × 41.9 × 20.3 cm^3^), maintained on a 12:12 h light/dark schedule, and provided access to food and water *ad libitum*. Animals were sustained on a minimum 10% fat diet and housed in a room kept at 24–26 °C in order to maximize thermogenic responses^47,48^. Animal maintenance and research were conducted in accordance with the eighth edition of the Guide for the Care and Use of Laboratory Animals as adopted and circulated by the National Institutes of Health, and protocols were approved by the Bowling Green State University Animal Care and Use Committee. All animals were allowed to acclimate to the facility for one week prior to the start of any treatments.

#### Drugs and chemicals

Racemic MDMA was donated by Dr. Mathew Banks of Virginia Commonwealth University (Richmond, VA) in the HCl salt form. Triamterene (TM) in salt form was obtained from Cayman Chemicals (Ann Arbor, MI), and iopanoic acid (IOP) in salt form was obtained from Fisher Scientific (Waltham, MA). All other chemicals and reagents were obtained from Sigma Chemical (St. Louis, MO). MDMA was diluted in normal saline (NS), and TM and IOP in dimethylsulfoxide (DMSO).

### ABX Study Design

Animals were randomly assigned to four treatment groups (n = 6): H_2_O saline, ABX saline, H_2_O MDMA, and ABX MDMA. Antibiotics were administered via the drinking water for fourteen days in accordance with the methods described in the study by Kiraly *et al*.^26^. Antibiotic doses were: Neomycin 2 mg/mL, Vancomycin 0.2 mg/mL, and Bacitracin 0.5 mg/mL. Fluid intake was measured daily, and body weight was measured every five days. On the fourteenth day, animals were injected subcutaneously with either saline or MDMA (20 mg/kg). Temperatures were taken rectally just prior to treatment (baseline) and every 30 min for an hour post-treatment using a Physiotemp Thermalert TH-8 termocouple (Physitemp Instruments, Clifton, NJ) attached to a RET-2 rectal probe. The animals were then euthanized by carbon dioxide asphyxiation and BAT, SKM, and cecum were dissected out for qPCR analysis and for bacterial count (cecum only), UCP 1&3 and TGR5 quantification (BAT and SKM only). To rule out the systemic effects of the antibiotics, six animals were injected intraperitoneally with 1.67 mg/kg vancomycin, 20 mg/kg neomycin, and 293 U/kg bacitracin in PBS^26^ thirty minutes before the administration of MDMA (20 mg/kg). Temperatures were recorded just prior to antibiotic administration (baseline) and every 30 minutes for an hour post-treatment of MDMA.

### RNA isolation and qRT-PCR

SKM and BAT were collected and preserved at −80 °C. Total RNA was isolated after homogenizing the tissues using PureZOL^™^ RNA Isolation reagent (Biorad). The concentration and quality of the RNA was determined using a NanoDrop Spectrophotometer (Thermo) and by 1% agarose gel electrophoresis, respectively. cDNA was synthesized from 200 ng of total RNA using the iScript™ Select cDNA Synthesis Kit (Biorad). Single-plex real-time quantitative PCR was carried out in CFX Connect Real-Time PCR Detection System (Biorad) using iTaq™ universal SYBR® Green supermix (Biorad) with the following parameters: 3 min at 95 °C; 40 cycles of 95 °C for 10 s, 52–58 °C for 30 s, 68 °C, 10 s; and graded heating to 95 °C to generate melt peak curve. Quantification cycle (Cq) values for all the genes within all four groups were compared and analyzed by using the ∆∆C(t) method^26^. All the primer pairs used for the analysis of *UCP1*, *UCP3*, *TGR5* and actin are shown in Table 1.

**Table 1:** Primers used for quantitative PCR reactions.

Target name	Forward primer (5′-3′)	Reverse primer (5′-3′)
Beta-actin	CAACCTTCTTGCAGCTCCTC	TTCTGACCCATACCCACCAT
UCP-1	ATCACCTTCCCGCTGGAC	GGCAGACCGCTGTAGAGTTTC
UCP-3	TGGTGAAGGTCCGATTTCAAG	CGTTTCTTGTGATGTTGGGC
TGR-5	CTGGCCCTGGCAAGCCTCAT	CTGCCATGTAGCGCTCCCCGT
EubF-16S	TCCTACGGGAGGCAGCAGT	GGACTACCAGGGTATCTAATCCTGTT

### qPCR for cecal bacteria

200 mg of frozen cecal samples from each animal were ground in liquid nitrogen and bacterial DNA was extracted using Puregene® Blood Core Kit B(Qiagen, CA) according to the manufacturer’s instructions. The concentration and quality of the DNA was assessed using NanoDrop (Thermo Fisher Scientific, PA). Equal volumes of 100 µl of DNA samples from each individual were pooled together into one sample for each treatment and was subjected to qPCR. Each qPCR reaction contained 200 ng of DNA in 20 µl reaction volume. The qPCR reaction (iTaq™ Universal SYBR® Green Supermix, BioRad, CA) contained the universal eubacterial primers for 16S rRNA gene listed in Table 1. The qPCR cycle parameters were 3 min at 95 °C; 40 cycles of 95 °C for 10 s, 55 °C for 20 s, and 68 °C, 10 s. Each qPCR reaction was run in triplicate along with reactions without DNA (no template) as negative controls. Quantification cycles (Cq) threshold was placed above baseline of the background noise of the fluorescence dye present in negative controls within the linear portion of the curve and were further used for statistical analysis.

### Agar Plate Assay and Antibiotic Screening

Cecal contents from all animals were collected in sterile tubes and adjusted to 200 mg/ml with 4 °C phosphate buffered saline (PBS; 0.1 M, pH 7.4). The resultant cecal slurries were homogenized and centrifuged at 400 × g for 2 minutes^49^. Serial 10-fold dilutions of the cecal slurry were spread on LB agar (Miller formulation; Difco-BRL) with or without 50 µg/ml kanamycin (LB_kan50_), incubated at 37 °C overnight, then manually scored for colony forming units (CFU), with the percent of kanamycin resistant (kan^r^) determined by dividing the number of CFU on the LB_kan50_ by the number of CFU on LB, and multiplying by 100.

### Isolation and Identification of Swarming Bacteria

Swarmers were isolated from the initial LB agar plates by subculture onto MacConkey agar (Difco-BRL), where the presence of bile salts inhibited swarming behavior. Plates were incubated for 18 hrs at 37 °C. Resultant colonies were picked and streaked for isolation onto fresh MacConkey agar and incubated as above. Isolated swarmer colonies were subjected to colony PCR, with the 16S rRNA gene amplified using the primer set Eub-16S F (Table 1)^50^ corresponding to bp of 331 to 799 of 16S rRNA of *Escherichia coli*. Sequencing of the PCR amplicons was performed at the University of Chicago DNA sequencing and genotyping facility, Chicago, Illinois, USA. Sequences were evaluated for similarity to known sequences in the National Center for Biotechnology Information (NCBI) nucleotide sequence database using BLAST^51^.

Because the resultant rRNA sequences were identical to those of both *P*. *mirabilis* and *S*. *enterica*, the phenotype of the swarming isolate was compared to that of *S*. *enterica*, by plating on the differential medium Hektoen enteric agar (Difco-BRL) and by the ability to swarm on LB agar.

### Pharmacological inhibition of TGR5 and D2 Study Design

Animals were randomly allocated into one of six test groups (n = 6): VEH/SAL (vehicle/saline), VEH/MDMA (MDMA only), TM/SAL (Triamterene only), TM/MDMA, IOP/SAL (Iopanoic acid only), and IOP/MDMA. Following the methods of Li *et al*.^24^, TM (50 mg/kg) was administered via an ip injection 30 minutes before treatment with MDMA (20 mg/kg, sc). Animals in the IOP/SAL and IOP/MDMA groups were pretreated with IOP (50 mg/kg/day, ip) daily for seven days, with the IOP dose size in accordance with Larsen *et al*.^25^. On day 7, animals were treated with MDMA (20 mg/kg, sc) 30 minutes after treatment with IOP or its vehicle, and core rectal temperature was measured at 30-minute intervals for 90 minutes post-treatment.

### Statistical analysis

GraphPad InStat v.6.0 software was used to complete all statistical analyses of data. Temperatures between treatment groups were compared using one-way ANOVA with a Student-Newman-Keuls post-hoc test. Additionally, temperatures within a treatment group were compared using a one-way ANOVA with a Dunnett’s post-hoc test. Maximum temperature change (ΔT_max_) was calculated by comparing the maximum increase in core temperature to the animal’s baseline temperature. An unpaired, two-tailed t-test was used to compare the maximum temperature changes between the ABX MDMA and H_2_O MDMA groups. Significance was set at the 95% confidence level (p < 0.05).
